# Manipulating Microbiota to Treat Atopic Dermatitis: Functions and Therapies

**DOI:** 10.3390/pathogens11060642

**Published:** 2022-06-02

**Authors:** Md Jahangir Alam, Liang Xie, Yu-Anne Yap, Francine Z. Marques, Remy Robert

**Affiliations:** 1 Department of Microbiology, Biomedicine Discovery Institute, Monash University, Clayton, VIC 3800, Australia; jahangir.alam@monash.edu (M.J.A.); liang.xie1@monash.edu (L.X.); 2 Hypertension Research Laboratory, School of Biological Sciences, Monash University, Clayton, VIC 3800, Australia; francine.marques@monash.edu; 3 Department of Physiology, Biomedicine Discovery Institute, Monash University, Clayton, VIC 3800, Australia; yu-anne.yap@monash.edu; 4 Heart Failure Research Laboratory, Baker Heart and Diabetes Institute, Melbourne, VIC 3004, Australia

**Keywords:** atopic dermatitis, skin microbiota, gut microbiota, metabolites, short-chain fatty acids, G-protein-coupled receptors, aryl hydrocarbon receptors, histone deacetylases, toll-like receptors, fecal microbiota transplantation

## Abstract

Atopic dermatitis (AD) is a globally prevalent skin inflammation with a particular impact on children. Current therapies for AD are challenged by the limited armamentarium and the high heterogeneity of the disease. A novel promising therapeutic target for AD is the microbiota. Numerous studies have highlighted the involvement of the skin and gut microbiota in the pathogenesis of AD. The resident microbiota at these two epithelial tissues can modulate skin barrier functions and host immune responses, thus regulating AD progression. For example, the pathogenic roles of *Staphylococcus aureus* in the skin are well-established, making this bacterium an attractive target for AD treatment. Targeting the gut microbiota is another therapeutic strategy for AD. Multiple oral supplements with prebiotics, probiotics, postbiotics, and synbiotics have demonstrated promising efficacy in both AD prevention and treatment. In this review, we summarize the association of microbiota dysbiosis in both the skin and gut with AD, and the current knowledge of the functions of commensal microbiota in AD pathogenesis. Furthermore, we discuss the existing therapies in manipulating both the skin and gut commensal microbiota to prevent or treat AD. We also propose potential novel therapies based on the cutting-edge progress in this area.

## 1. Introduction

Atopic dermatitis (AD), also known as eczema, is a skin inflammation that exhibits chronic, persistent, pruritic lesions, and is often associated elevated levels of IgE [1,2]. AD affects 10–20% of the population during their lifetime in developed countries, with a particularly high prevalence among children [2,3]. Its prevalence is also rapidly increasing in developing nations [4,5]. The established pathogenesis of AD involves the initiation of barrier disruption, followed by the activation of type 2 (T_H_2) immune responses [2,6]. Variants in the filaggrin (*FLG*), the gene encoding an important skin barrier protein, represent a significant risk factor for AD [7,8,9]. AD is often associated with the development of asthma and food allergies, which is known as “atopic march” [10]. Physical therapies that moisturize the skin, preventing water loss, controlling xerosis, and relieving barrier disruptions, are recommended for AD patients [11]. While corticosteroids remain the standard anti-inflammatory treatment against AD, the efficacy of blocking T_H_2 responses is recognized by various clinical trials [12,13,14,15,16,17,18,19]. Unfortunately, the limited armamentarium [20] and the high heterogeneity of the disease [21] make the management of AD challenging. Therefore, novel therapeutic strategies needed for AD treatment.

The past two decades have highlighted the role of commensal microbiota in health homeostasis and disease [22]. As the largest and outmost organ of the body, the human skin has been estimated to host about 1 billion bacteria per 1 cm^2^ area [23]. Conversely, the gastrointestinal tract harbors the largest microbiota population in our body, exceeding 10^14^ bacterial cells [24,25]. AD is probably the most well-characterized disease in which skin dysbiosis plays a causal role [6]. In addition, AD is also associated with gut dysbiosis [26]. The investigations from the pre-clinical animal studies and the emerging human 3D skin models furthered our understanding on the complex interplay between the microbiota and the AD context [27,28]. In this review, we comprehensively summarize the roles of both the skin and gut microbiota in AD and the current approaches aimed at manipulating commensal microbiota to prevent or treat AD. We also provide insights for future investigations to improve the efficacy of current agents and establish novel therapeutic strategies that leverage the microbiota.

## 2. The Skin Microbiota Alternation in AD Patients: A Particular Focus on *Staphylococcus aureus*

It is now well established that changes in the normal skin microbiota composition, a condition known as dysbiosis, contribute to the disruption of cutaneous immune homeostasis and promotes the development of skin diseases, including AD [29,30]. Studies that have demonstrated the association of AD with skin dysbiosis are summarized in Table 1. AD is often accompanied with dysbiosis, featured by increased colonization of staphylococcal species and decreased richness and diversity of other bacterial communities [31]. *S. aureus*, a dominant species among the family of Staphylococcae, can be 100 times more abundant in AD skin compared to normal healthy skin [32]. On the skin surface, *S. aureus* secretes virulence factors, including phenol-soluble modulins (PSMs) and proteases, thus disrupting normal skin barrier functions, which alter the epidermal environment favouring the development of AD [33,34,35,36,37]. We will discuss the mechanisms involved later in this review.

The increased abundance of *S. aureus* colonization in AD is associated with a depletion in the coagulase-negative staphylococcal species (CoNS), such as *S. epidermidis*, *S. hominis*, and other skin commensal bacterial communities, including *Streptococcus salivarius*, *Propionibacterium*, *Streptococcus*, *Acinetobacter*, *Corynebacterium*, *Prevotella* and Proteobacteria [31,38,39,40]. In contrast, these skin commensal microbiota produce antimicrobial substances that inhibit the growth of pathogenic *S. aureus* and its biofilm formation [30,41,42]. In the non-lesional skin samples of AD patients, CoNS are the dominant bacterial communities [38]. However, their abundances were lower on the skin of healthy individuals compared with non-lesional AD skin [38,43]. This suggests the existence of complex interactions between the different skin microbial communities, which modulate the host’s susceptibility to AD.

**Table 1:** Summary of the studies demonstrating the dysbiosis of skin microbiota in AD.

Year	Subjects, Numbers	Methods	Results (Alternations of Skin Microbiota)	Reference
2012	11 Infants with AD and 12 healthy controls	16S rRNA gene sequencing	AD infants: ↓*S. salivarius* and ↑*S. aureus*	[40]
2012	12 Children with AD and 11 healthy controls	16S rRNA gene sequencing	AD lesion: ↑*S. aureus* and ↑*S. epidermidis*	[31]
2013	13 AD patients and 49 healthy controls	16S rRNA gene sequencing	AD patients: ↑*S. aureus*, ↓-diversity	[44]
2015	21 AD infants and 17 healthy controls	Real-time PCR analysis of skin scratches	AD infants: ↑*S. aureus*	[45]
2016	128 AD patients (59 young children at 2–12 years, 13 teenagers at 13–17 years, and 56 adults at 18–62 years of age), 68 age-matched healthy controls (13 young children, 10 teenagers, 45 adults)	16S rRNA gene sequencing	Children with AD: ↑*Streptococcus*, ↑*Granulicatelle*, ↑*Gemella*, ↑*Rothia*, ↑*Haemophilus* Adults with AD: ↑*Propionibacterium*, ↑*Corynebacterium*, ↑*Staphylococcus*, ↑*Lactobacillus*, ↑*Finegoldia*, ↑*Anaerococcus*	[46]
2016	Three male first cousins aged 50–53 years	16S rRNA gene sequencing	AD patients: ↑*S. aureus*	[47]
2017	10 AD infants, 10 age-matched healthy controls	16S rRNA gene sequencing	AD infants: ↑*Staphylococcus*	[29]
2017	49 AD patients and 30 non-AD subjects	16S rRNA gene sequencing	AD patients: ↓*S. epidermidis*, ↓*S. hominis*	[30]
2017	27 AD patients and 6 healthy controls	High-throughput pyrosequencing	AD patients: ↑*Staphylococcus*, ↑*Pseudomonas*, and ↑*Streptococcus*, ↓*Alcaligenaceae* (*f*), ↓*Sediminibacterium*, and ↓*Lactococcus*	[48]
2018	10 AD patients and 10 healthy controls	16S rRNA gene sequencing	AD patients: ↑*S. aureus*, ↓-diversity	[38]
2019	91 AD patients, 134 psoriasis patients, and 126 healthy controls	16S rRNA gene sequencing	AD patients: ↑*S. aureus*, ↓*S. epidermidis* and *↓Corynebacterium*	[49]
2019	172 AD patients and 120 healthy controls	16S rRNA gene sequencing	AD patients: ↑*Staphylococcus*	[50]
2020	11 AD patients	16S rRNA gene sequencing	AD skin lesions: ↑*S. aureus*, ↓*C. pseudogenitalium*	[51]
2020	67 AD patients and 28 healthy controls	16S rRNA gene sequencing	AD skin lesion: ↑*Staphylococcus* (*S. aureus* and *S. epidermidis*), ↓*Corynebacterium*, ↓*Micrococcus*, ↓*Cutibacterium* and ↓*Streptococcus*	[52]
2020	7 AD patients and 10 healthy controls	16S rRNA gene sequencing, and *Staphylococcus* specific SLST sequencing	AD patients: ↑*Staphylococcus*, ↓*Propionibacterium*	[53]
2021	28 AD patients and 14 healthy controls	16S rRNA gene sequencing	AD patients: ↑*S. aureus*, ↓*S. capitis* and ↓*Micrococcus* sp.	[54]

Legend: SLST, single-locus sequencing typing.

## 3. *S. aureus* in AD Pathogenesis

Probably the best understood association between skin microbiota and AD is the involvement of *S. aureus*. As previously discussed, *S. aureus* is commonly identified in the skin of AD patients [34,55,56]. *S. aureus* colonization precedes the onset of AD in infancy [57], and topical application of *S. aureus* isolates from AD skin suffices to induce AD-like skin inflammation in mice [27]. This evidence supports the causal role of *S. aureus* in AD pathogenesis. *S. aureus* adheres to AD skin biopsy specimens more efficiently compared to healthy ones because of the changes to the composition of the stratum corneum and corneocyte morphology [58,59]. The impairment of the skin barrier during AD facilitates the colonization of *S. aureus* [60]. Filaggrin deficiency is associated with elevated *S. aureus* in skin microbiota [43]. Reduced filaggrin expression promoted *S. aureus* colonization in a human 3D epidermal model [61]. T_H_2 cytokines such as IL-4 and IL-13 can suppress the production of antimicrobial peptides (AMPs) such as cathelicidin and β-defensins that can act against *S. aureus*, hence promoting its excessive expansion [62]. Additionally, *S. aureus* proteases cause epidermal disruption [33,63]. *S. aureus* stimulates keratinocytes to produce endogenous proteases, which exacerbate the barrier dysfunctions [37]. Furthermore, *S. aureus* produces a cysteine protease staphopain and a metalloprotease aureolysin that cleave and inactivate the AMPs [62,64].

*S. aureus* expresses a variety of virulence factors inducing inflammation related to AD [65,66,67,68]. For example, *S. aureus* expresses superantigens (SAgs), such as toxic shock syndrome toxin-1 (TSST-1) and the staphylococcal enterotoxin serotypes [67]. These SAgs bind to major histocompatibility class II (MHC-II) and stimulate the production of cytotoxic cytokines from T cells [69,70,71,72]. In addition, SAgs trigger IgE response, induce mast cell degranulation, and promote skin inflammation [73].

δ-toxin and α-toxin are two other major virulent factors secreted by *S. aureus* [66,68]. Enhanced by IgE, δ-toxin induces degranulation without lysis of murine mast cells [73]. *S. aureus* lacking δ-toxin was not able to induce skin inflammation characterized by elevated IL-4 and IgE in mice [73]. Phenol-soluble modulin (PSM) including PSMα and PSMβ are short amphipathic peptides expressed by *S. aureus* with similar functions as δ-toxin [68]. By opposition to δ-toxin, PSMα2 and PSMα3 cause mast cell death [73]. PSMα also induces proinflammatory cytokines production from keratinocytes, including IL-1α and IL-36α, which stimulates γδ T cells and elicit Th17 responses as well as neutrophil recruitment [36,74,75]. α-toxin is a pore-forming cytolysin, which can induce skin inflammation in mice [66,76]. The keratinocyte death caused by α-toxin directly exacerbates the barrier disruption, thus promoting AD-like inflammation [77]. The keratinocyte toxicity is enhanced by T_H_2 cytokines during AD. Meanwhile, under a healthy state, the expression of filaggrin and sphingomyelinase reduces keratinocyte susceptibility to α-toxin [78]. Protein A found on the cell wall of *S. aureus* binds to the tumour necrosis factor receptor 1 (TNFR1) and induces NF-κB and AP-1 activation and their downstream proinflammatory pathways in keratinocytes [79].

Furthermore, *S. aureus* can enter from the epidermis to the dermis, where it encounters immune cells and triggers the T_H_2 responses by inducing the production of IL-4, IL-13, IL-22, thymic stromal lymphopoietin (TSLP), and other cytokines associated with AD [63]. The pathogen-associated molecular patterns (PAMPs) from *S. aureus* predominantly bind to toll-like receptor 2 (TLR2) [80,81]. TLR2 signalling is a double-edged sword in AD pathogenesis. Although TLR2 activation stimulates TSLP production and mast cell degranulation [73,82,83], it can also be protective in AD by rapidly increasing the expression of tight junction protein claudin 1 and AMPs in differentiated epidermal layers from normal skin [84]. AD skin has impaired TLR2 activity, thus disrupting the normal immune response and skewing T_H_2 immunity [84,85].

## 4. Other AD Pathogeneses Regulated by the Skin Microbiota

**Figure 1:**
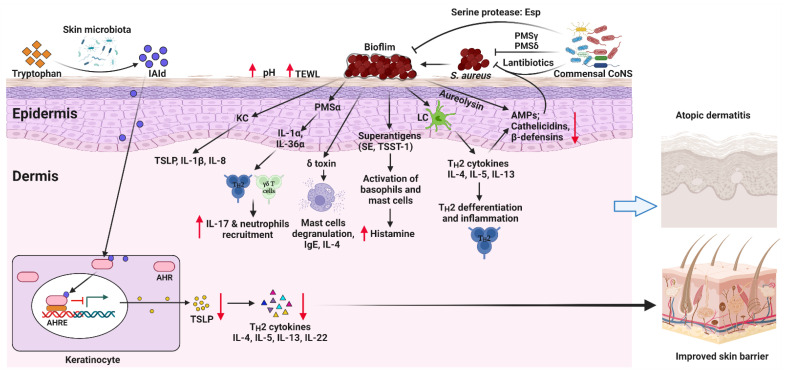
Mechanisms of how skin microbiota regulates AD pathogenesis. Excessive *S. aureus* colonization on the skin leads to the formation of biofilms and the secretion of virulence factors. These virulence factors facilitate mast cell degranulation, enhance inflammatory cytokine productions and the release of histamine, and increase IgE levels. In addition, *S. aureus* directly stimulates keratinocytes (KC) and Langerhans cells (LC) to release proinflammatory cytokines, including TH2 cytokines, TSLP, IL-8, and IL-1β, thus inducing TH2 differentiation and inflammation. Together, excessive cutaneous *S. aureus* colonization promotes AD pathogenesis. Conversely, commensal Coagulase-negative staphylococci (CoNS) inhibit the colonization of *S. aureus* by producing lantibiotics, PSMγ, and PSMδ. They also inhibit *S. aureus* biofilm formation by producing the serine protease glutamyl endopeptidase (Esp). Tryptophan metabolites derived from skin microbiota can activate AHR, thus inhibiting TSLP production by KCs and improving the epidermal barrier of the skin. AHR, aryl hydrocarbon receptor; AHRE, AHR element; AMPs, antimicrobial peptides; CoNS, Coagulase-negative staphylococci; IAId, indole-3-aldehyde; IL, interleukin; ILC3, lymphoid cells type 3; KC, keratinocyte; LC, Langerhans cell; PSM, phenol-soluble modulin, TSLP, thymic stromal lymphopoietin; SE, Staphylococcal enterotoxin; TSST-1, toxic shock syndrome toxin-1. Created with BioRender.com (accessed on 4 May 2022).

In addition to the pathogenic role of *S. aureus*, homeostasis of skin microbiota is vital for normal skin barrier function, the cutaneous immune balance, and the elimination of possible pathogens, thus modulating AD pathogenesis [86]. Germ-free mice exhibited a significantly different skin transcriptome [87]. Germ-free mice displayed impaired skin barrier function, a direct consequence of the abnormal epithelial development and differentiation [88]. Treating a 3D skin tissue model with a mix of selected members of normal skin microbiome profoundly improved the barrier integrity of the tissue [89]. In this section, we discuss how the skin microbiota regulates two major aspects: (1) AMPs, and (2) the tryptophan metabolites-aryl hydrocarbon receptor (AHR) axis. Together with the pathogenic roles of *S. aureus* in AD, these mechanisms are summarized in Figure 1.

### 4.1. Antimicrobial Peptides

Using antimicrobial mechanisms [90], the skin barrier and its associated microbiota protect against pathogenic microorganisms such as *S. aureus* in AD. For example, the production of AMPs such as cathelicidin and β-defensin by the host and the microbiota inhibit certain microorganisms. It appears that normal commensal bacteria are more resistant to host AMPs [91], and host production of AMPs is regulated by commensal bacteria in poorly understood mechanisms [92,93,94]. Not only can commensal bacteria control the production of AMPs by the host, they also produce some antimicrobial agents able to suppress pathogenic competitors, thereby providing an additional antimicrobial barrier on the skin. For instance, *S. epidermidis* produces the peptides PSMγ and PSMδ, which inhibit the growth of pathogenic bacteria on the skin [95,96]. Similarly, *S. hominis* is another CoNS that produces lantibiotics, a class of cyclic AMPs containing lanthionine and methyllanthionine [30]. Skin dysbiosis likely leads to the dysregulation of the antimicrobial function against pathogenic bacteria. Impaired production of cathelicidin and β-defensins is responsible for the dominance of *S. aureus* in AD skin [62]. Therefore, the skin microbiota has an interaction with the host AMPs production and contributes to the control of AD.

### 4.2. Tryptophan Metabolites-AHR Axis

AHR is a ligand-activated transcription factor that modulates tissue homeostasis and immune responses [97,98,99]. AHR agonists range from xenobiotic chemicals to endogenous indole by-products derived from tryptophan metabolism [100,101,102]. The activation of AHR is mostly anti-inflammatory through the regulation of cytokine production and other immune-related transcription factors, such as NF-κB [97,98,99]. AHR is expressed by keratinocytes, epidermal Langerhans cells, dermal and epidermal innate and adaptive immune cells [103]. Although T_H_2 cells exhibited negligible AHR expression [104], activation of AHR in dendritic cells (DCs) suppressed T_H_2 differentiation [105,106]. The topical application of coal tar, which is a traditional therapy against AD, works by activating AHR [107]. In the skin epidermis, dead keratinocytes and broken keratin are used as substrates in the tryptophan metabolism of skin microbiota which leads to AHR agonists production [108]. The barrier dysfunction observed in germ-free mice skin is attributed to the attenuated AHR pathway [88]. Activation of AHR upregulates the expression of barrier-related molecules like filaggrin, loricrin and involucrin, by keratinocytes, thus maintaining the healthy epidermal barrier [109,110,111]. This can be achieved by the tryptophan metabolites derived from the skin microbiota [111,112,113,114]. For example, Indole-3-aldehyde (IAld), a skin microbiota-derived AHR agonist, suppresses TSLP expression and protects mice against MC903-induced AD. In humans, the skin of AD patients displays a lower level of IAld compared to that of their healthy counterparts [113]. AHR signalling is essential for AMPs production and can shape the skin microbiome [53], as demonstrated with AHR-deficient mice, which display a more variable and complex skin microbiota compared to WT controls [115].

## 5. Therapeutic Manipulation of the Skin Microbiota in the Management of AD

Reducing the abundance of pathogenic bacteria and restoring the normal microbial balance in the skin may be of clinical benefit for AD management. In animal models, antibiotic treatment established the causal role of skin dysbiosis in AD. Although a randomized clinical trial (RCT) has demonstrated that antibiotics suppressing the growth of *S. aureus* significantly attenuated AD [116], the clinical efficacy of antibiotics in this pathology remains questionable [117]. Skin bacteria abundantly reside in hair follicles, eccrine glands or beneath the epidermal barrier, where antibiotics are difficult to reach [63,118]. In addition, most antibiotics can (1) disrupt normal microbiota due to their lack of specificity and (2) favour antibiotic-resistant strains of *S. aureus*. Nevertheless, a phase III trial (NCT02840955) investigates the therapeutic potential of a bacteriophage endolysin (Staphefekt) in AD adult patients. Staphefekt is a specific bactericidal agent against *S. aureus* with unlikeliness of resistance and is effective against the methicillin-resistant *S. aureus* (MRSA) strains that have already acquired resistance to conventional antibiotics. Because bacterial killing by this endolysin is independent of the involvement of the bacterial metabolism [119,120,121].

The reintroduction of normal commensal bacteria represents a promising strategy for AD treatment. Topical application of antimicrobial CoNS strains able to produce specific AMPs against *S. aureus*, decreased colonization of this bacteria and effectively attenuated AD severity [30,122]. Another angle in manipulating skin microbiota is by reducing the skin surface pH. The acidic epidermal surface is essential for maintaining the skin barrier [123]. Acidity favours commensal bacteria and suppresses pathogenic bacteria, including *S. aureus* [124]. In healthy individuals, it has been observed that higher skin pH was associated with increased *S. aureus* skin colonization [125]. It is well known that AD lesions display higher pH values than healthy skin [126,127], and topical application of acids improved AD in murine models [128,129].

Current attempts to manipulate skin microbiota to control AD are limited and lack RCTs. However, as more causal roles of the skin microbiota in AD are being revealed, therapeutic manipulation of skin microbiota emerges as a promising way to improve the management of AD.

## 6. The Gut Microbiota Profiles in AD Patients

Aside from the skin microbiota, the gut microbiota, known to have a systemic impact on the host immune responses, is also closely associated with atopic diseases such as AD. A hypothesis of the “gut-skin” axis has been proposed and has opened new paths related to the prevention and treatment of AD [26]. Various studies demonstrated that AD is associated with gut dysbiosis, especially during early life (Table 2). AD patients display poor gut microbial diversity in several clinical trials [130,131,132,133,134,135,136,137,138], while contradictory results exist [139,140,141,142]. Similarly, to the skin, AD patients exhibit abundant *S. aureus* in their gut microbiota [143,144,145,146]. Other microbes associated with inflammation and epithelial damage, such as *Clostridiodes difficile*, and coliforms, including pathogenic *Escherichia coli*, are increased in the gut microbiota of AD patients [136,140,146,147,148,149,150,151,152]. Metagenomic analysis has shown that the gut microbes in AD patients carry extra genes associated with inflammatory responses and the breakdown of gut epithelial layers [139,141]. Breastfed infants have a lower risk of atopic diseases [153,154,155,156,157,158], likely because of the abundance of *Bifidobacteria* in their gut [159,160,161,162], a genus that is reduced in the gut microbiota of AD patients [151,163,164,165,166]. However, it has been reported that AD development is also associated with the higher abundance of certain species of *Bifidobacteria*, such as *Bifidobacterium catenulatum*, *B. bifidum* and *B. pseudocatenulatum* [167,168]. Short-chain fatty acids (SCFAs) are microbial metabolites well known for their anti-inflammatory effects. High fecal levels of SCFAs are significantly associated with a lower risk of AD [139,169]. The SCFA-producing bacteria *Coprococcus eutactus* is less abundant in the gut microbiota of AD patients [135]. Subspecies of *Faecalibacterium prausnitzii* are enriched in AD fecal samples. Although *F. prausnitzii* is a major SCFA producer in healthy subjects, this species is an inefficient SCFA producer in AD patients [139]. Furthermore, genes encoding carbohydrate active enzymes (CAZymes), which break down resistant starch into SCFAs, are deficient in the gut microbiota of AD patients [142]. While infantile gut dysbiosis may predict the development of AD [131,132,134,147,148,149,152], childhood AD history had prolonged imprints in gut microbiota that neonates with childhood AD history had a lower abundance of *Bifidobacteria*, *Akkermansia*, and *Faecalibacteria* compared to their healthy counterparts [170].

Despite these reports linking gut dysbiosis with AD, the actual roles of gut microbiota in AD development are still largely unknown. Establishing a causative role for the gut microbiota in the development of diseases remains challenging. An invaluable tool for this type of investigation is the use of germ-free animals. Germ-free mice showed similar disease severity in the oxazolone-induced ear atopic dermatitis model as conventional mice [171]. However, germ-free mice have higher levels of serum IgE and inflammatory cytokines, such as TNF and IL-6 in ear tissues, suggesting exacerbated skin inflammation [171]. Furthermore, the sensitivity to oxazolone is transferable to germ-free mice with fecal microbiota transplant (FMT) [171]. These findings suggest a causative role of gut microbiota in the development of AD. However, the direct link between gut microbiota and type 2 inflammatory responses in AD is still unknown.

## 7. The Gut Microbiota Regulates AD-Related Immune Responses and the Underlying Mechanisms

**Figure 2:**
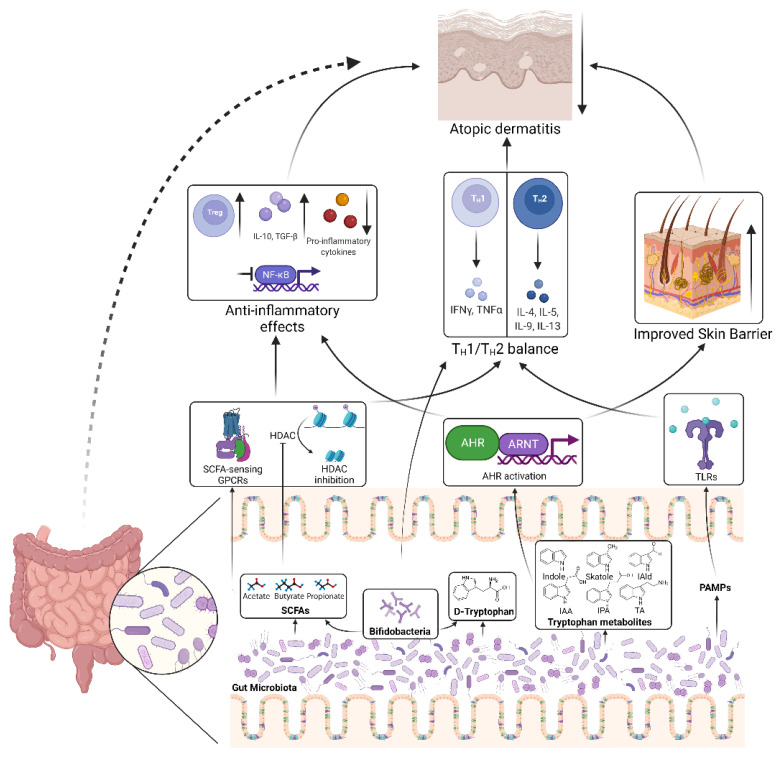
Mechanisms of how gut microbiota regulate AD pathogenesis. Short-chain fatty acids (SCFAs) produced by the gut microbiota are able to activate SCFA-sensing G-protein coupled receptors (GPCRs) and/or inhibit histone deacetylases (HDACs), thus activating downstream signalling cascades that suppress inflammatory responses and restoring TH1/TH2 balance. Microbial metabolite, D-tryptophan can also restore TH1/TH2 balance. *Bifidobacteria*, a genus of bacteria to which many probiotics belong, are an important source of these metabolites. Microbial tryptophan metabolites can activate the aryl hydrocarbon receptor (AHR), which inhibits inflammatory responses and improve the epidermal barrier of skin. Pathogen-associated molecular patterns produced by the gut microbiota can activate toll-like receptors (TLRs) thus restoring TH1/TH2 balance. All these mechanisms benefit AD. IAld, indole-3-aldehyde; IAA, indole-3-acetic acid; IPA, indole-3-propionic acid; TA, tryptamine; ARNT, aryl hydrocarbon receptor nuclear translocator. Created with BioRender.com (accessed on 4 May 2022).

There is firm evidence supporting the role of gut microbiota-regulated immune responses in atopic diseases and AD [172]. For example, early colonization of *Bifidobacteria* subtly regulates the T_H_1/T_H_2 immune balance, reducing the risk of AD [173]. Most of the knowledge regarding this arises from the use of probiotics in different clinical trials, as discussed below. In this section, we focus on three aspects: (1) SCFAs-anti-inflammation axis, (2) tryptophan metabolites-AHR axis, and (3) toll-like receptor signalling (Figure 2). These pathways that can be modulated by gut microbiota are well characterized and highly relevant to AD and other allergic immune responses.

### 7.1. Short-Chain Fatty Acids and Their Anti-Inflammatory Effects

As briefly discussed above, the gut microbiota of AD patients contains less SCFA-producers or is defective in generating SCFAs [135,139,142]. SCFAs are microbial metabolites derived from dietary fibre [174]. The most abundant SCFAs found in the host are acetate (two carbons), propionate (three carbons), and butyrate (four carbons) [175]. Recent studies established a potent anti-inflammatory role of SCFAs against numerous inflammatory diseases, including atopic diseases [169,172,176,177,178]. In atopic models, the anti-inflammatory effects of SCFAs are promoted through the regulation of DCs leading to the inhibition of T_H_2 [179] and the activation of regulatory T cell (Treg) differentiation [180]. Subcutaneous injection and/or topical application of sodium butyrate attenuate the hapten-induced AD in mice by recruiting Tregs and inducing the production of the anti-inflammatory cytokine, IL-10 [181]. Still, the mechanisms underlying the protective effect of SCFAs in AD are poorly understood and insights from other atopic diseases would be invaluable.

The immune regulations by SCFAs are largely mediated by two key mechanisms: (1) the activation of certain G-protein coupled receptors (GPCRs) and (2) the inhibition of histone deacetylases (HDACs) [176].

#### 7.1.1. SCFA-Sensing GPCRs

There are three GPCRs activated by SCFAs. GPR41 and GPR43 are exclusively activated by all three abundant SCFAs, while GPR109A can only be activated by butyrate [182,183]. The role of GPR41 and GPR109A in the immune cells remains elusive, while more information is available about GPR43, which is more widely expressed by the immune cells compared to the other two receptors [182,184]. Activation of GPR41 and GPR43 inhibits the TNF-induced or LPS-induced production of the pro-inflammatory cytokines IL-6 and IL-8 in human endothelial cells [185]. GPR43 was the first SCFA-sensing receptor to be deorphanized. It plays an anti-inflammatory role in various disease models, including atopic diseases [180,184,186,187,188,189]. A report demonstrated that T cells recruited to the skin after butyrate administration showed higher expression of GPR43 [181]. GPR43 is essential for the expansion of Tregs and the regulatory effects of Tregs induced by SCFAs [190,191]. Activation of GPR43 promotes the production of the anti-inflammatory cytokine IL-10 by CD4^+^ T cells [192]. Regulation of neutrophil biology, which is involved in AD [193,194,195,196,197], by SCFAs is also dependent on GPR43 activation [184,198].

GPR109A can regulate macrophages and DCs to promote colonic Treg development [199]. Moreover, GPR109A signalling inhibits the production of the pro-inflammatory cytokines, TNF, IL-6, CCL2, and IL-1β from monocytes, macrophages, adipocytes, and epithelial cells [200,201,202,203]. However, the role of GPR109A in regulating T_H_2 cytokine production, particularly in the AD context, remains unclear. 

Conversely, SCFAs significantly reduce the luminal pH in the colon [204,205], thus inhibiting the survival of potential pathogens [206] and activating proton-sensing GPCRs of the host [172,182]. We recently reported that GPR65, a proton sensor highly expressed in various leukocyte subsets, plays a protective role in AD by inhibiting inflammatory cytokines production and immune cell migrations [207]. The polymorphisms resident in or near the human gene *GPR65*, rs3742704, and rs8005161 were significantly associated with the risk of AD and asthma [207,208].

#### 7.1.2. HDAC Inhibition

HDACs are enzymes that remove acetyl groups from an ε-N-acetyl lysine amino acid on histones [209]. The histone deacetylation facilitates high-affinity binding between histone proteins and DNA, leading to DNA compaction [209], results in lower chromatin accessibility for transcription factors and prevents gene expression [209]. HDACs play a critical role in many pathologies, including atopic diseases, making HDAC inhibitors promising drug candidates against atopic diseases and AD [210]. High HDAC activity impairs tight junction function [211]. HDAC inhibitors such as trichostatin A (TSA) inhibit 2,4-dinitrofluorobenzene-induced dermatitis in mice [212,213]. A recent study demonstrates that belinostat, a pan HDAC inhibitor, can rescue the defective skin barrier in AD through restoring epidermal miR-335 expression [214]. miR-335 directly represses SOX6, which impairs epidermal differentiation [214]. However, a report also demonstrated that HDAC inhibited IL-4 production by human T cells, and TSA promoted IL-4 production [215].

SCFAs, particularly butyrate, are potent inhibitors of HDACs [216,217,218,219,220,221]. SCFAs are able to inhibit HDACs directly by entering the cells [222] and this inhibition is closely associated with GPCR signalling. The activation of GPR41 can suppress histone acetylation in Chinese hamster ovary cell lines, possibly through inhibiting HDACs [223]. HDAC inhibition in colonic Tregs by SCFAs may be dependent on GPR43 [190]. SCFAs restrict pro-inflammatory cytokine production through inhibiting HDAC activity. Acetate reduced HDAC activity in human monocytes in vitro, which correlated with lower production of IL-6, IL-8, and TNF [224]. SCFAs showed a similar effect as TSA in suppressing NF-κB activation and TNF production in human peripheral mononuclear cells, but HDAC activity was not tested [225]. Similarly, HDAC inhibition can partially mediate SCFAs’ inhibition of pro-inflammatory cytokine production by human endothelial cells [185]. Whether SCFAs regulate T_H_2 cytokine production through HDAC inhibition remains unknown.

Conversely, HDAC inhibition may underlie Treg promotion by SCFAs. HDAC inhibitors induce Foxp3 expression and promote Treg differentiation [226,227,228]. The most well-known HDAC regulating Treg differentiation and function is HDAC9 [228], while inhibition or depletion of HDAC10 and HDAC11 also enhances the immune-suppressive function of Tregs [229,230]. *Hdac9*^−/−^ mice had ~50% more Foxp3^+^ Tregs in the lymphoid tissues, and *Hdac9*^−/−^ Tregs are also more potent in suppressing effector T cells compared to WT controls [228]. Overall, SCFAs promote immunity and suppress inflammatory responses associated with AD.

### 7.2. Tryptophan, Its Metabolites and Aryl Hydrocarbon Receptor Signalling

Tryptophan is an essential amino acid in human. While L-tryptophan represents the proteinogenic enantiomer, its D-enantiomer, D-tryptophan, is a bacterial metabolite from *Bifidobacterium*, *Lactobacillus* and *Lactococcus*, which are known probiotics [231]. A recent study demonstrated that D-tryptophan expands the Treg population in the colon [231], and suppresses the expression of T_H_2-associated CCL17 by the human Hodgkin lymphoma T-cell line KM-H2 and the inflammatory activity of human DCs [231].

Meanwhile, by breaking down dietary L-tryptophan into indole, indole-3-acetic acid (IAA), indole-3-propionic acid (IPA), tryptamine (TA), and 3-methyl indole (skatole) [232,233], the gut microbiota also serves as a vital source of AHR agonists. A landmark study demonstrated that the AHR agonist IAld produced from the tryptophan metabolism of *Lactobacillus reuteri* activates AhR, induces IL-22 secretion, and promotes gut homeostasis [234]. This suggests that AHR serves as the bridge in the crosstalk between commensal bacteria and host health. Knowing that AHR agonists, such as IAld, have potent protection against AD, we hypothesize that AHR agonists derived from gut microbiota have distal protection for AD in the skin.

### 7.3. Toll-like Receptor Signalling

In addition to skin microbiota, gut microbiota also produces PAMPs recognized by TLRs, thus contributing to systemic immune homeostasis [235], including AD-related immune responses. As mentioned earlier, TLR2 and TLR4 play essential roles in maintaining the balance between T_H_1 and T_H_2 immune responses, thus regulating AD symptoms [236,237,238,239]. Gut microbiota composition is associated with TLR signalling in atopic diseases. Infants with eczema have lower fecal *Ruminococcaceae*, which is negatively associated with TLR2-induced IL-6 and TNF production [240]. Moreover, TLR4 SNP rs10759932 and fecal *E. coli* had a significant multiplicative interaction regarding allergic sensitization [241]. To the best of our knowledge, few studies have looked at the crosstalk between gut microbiota and TLRs in AD development.

## 8. Therapeutic Manipulation of Gut Microbiota in the Prevention and Treatment of AD

Since gut microbiota is likely to regulate AD pathogenesis, both gut microbiota and microbial metabolites are promising tools in controlling AD prevalence. The major therapeutic methods relevant to gut microbiota include FMT, the use of prebiotics, probiotics, synbiotics, and postbiotics. These methods all aim to restore the balance of gut ecology in order to modulate allergic immune responses. While abundant trials attempted to use these as therapies to prevent and treat AD, at this stage, their efficacies remain inconclusive due to inconsistent results and potential risks.

### 8.1. Fecal Microbiota Transplantation

The most direct method to re-establish the balance of the gut microbiota is FMT. FMT has been successfully used to treat various diseases, including *Clostridium difficile* infection [242]. A recent study reported that FMT using feces from healthy BALB/c donors successfully attenuated ovalbumin-induced AD in BALB/c mice [243], hinting at the promise of FMT in AD management. However, caution should be taken when applying FMT. FMT has resulted in several deaths in the recent decade through the spread of multidrug-resistant bacteria, aspiration, and toxic megacolon [244,245,246,247] and has elicited an important safety alert from United States Food and Drug Administration in 2019 [244]. Due to the limited knowledge on the gut microbiota, the actual operative agents in FMT treatments are mostly unknown, and the uncertain risks are not well known. Since patients with gut dysbiosis tend to have compromised gut barrier, they are more susceptible to the risks associated with FMT. Furthermore, regulatory benchmarks and standardized protocols for FMT are still absent or at least limited in many jurisdictions [248]. The alternative microbiota-based therapies with rationally selected microorganisms or microbial metabolites may be preferable for future AD treatments.

### 8.2. Prebiotics in Preventing and Treating AD

Prebiotics are non-viable substances that are selectively utilized by host microorganisms conferring health benefits [249]. Prebiotics are naturally rich in human milk and vegetarian food, including cereal, fruits, and vegetables. Modern industry also produces some prebiotics. Therefore, prebiotics can be supplemented either directly or by modifying food intake.

Investigations into the health effects of breastfeeding on infants led to the first applications of prebiotics to benefit health. Breastfed infants have different fecal bacterial composition compared to non-breastfed infants, with enrichment of *Bifidobacteria*, and such difference correlates with the health status of the infants [159,160,161,162]. Recent studies confirmed that intake of breast milk is the most significant factor shaping the early life gut microbiota [250,251]. Breastfed infants have a reduced risk of childhood AD compared to non-breastfed infants [153,154,155,156,157,158]. The beneficial effect of breast milk has been attributed to the prebiotics, human milk oligosaccharides (HMOs) [252,253,254]. HMOs selectively expand *Bifidobacteria*, particularly *B. bifidum* and *B. longus*, which, together with *B. breve* are the most abundant bacteria in the gut microbiota of breastfed infants [255]. Supplementing HMO effectively modulates the infant gut microbiota [256]. However, whether supplementing breast milk-derived prebiotics prevents AD is still questionable. Supplementing HMOs, mostly galacto-oligosaccharide (GOS) and fructo-oligosaccharide (FOS), effectively reduced the incidence of AD during infancy but exhibited poor long-term protection in several RCTs [257,258,259,260,261,262,263,264]. The results from other trials were not sufficiently in favour of supplementing HMOs in treating AD [265,266,267,268,269,270,271]. Of note, no adverse side effects were noted in all these trials and prebiotic HMOs have been added to infant milk formula to mimic the bifidogenic effect of breast milk [258,272,273].

Fermentable dietary fibres are probably the most popular prebiotics in recent biomedical studies [249]. They can be fermented by the commensal gut microbiota into SCFAs, whose anti-inflammatory functions have been discussed above. However, whether supplementing fermentable dietary fibre would benefit AD still requires more investigations in both pre-clinical and clinical studies.

Due to the lack of solid scientific evidence, the use of prebiotics is not recommended by World Allergy Organization (WAO) as a preventive or therapeutic measure against AD [270]. Future studies may demonstrate the efficacy of using non-digestible carbohydrates in preventing or even treating AD.

### 8.3. Probiotics in Preventing and Treating AD

Probiotics are living microorganisms that confer a health benefit on the host when administered in an adequate amount [274]. The benefits of probiotics in AD are studied more extendedly and thus have more solid conclusions. The most commonly used probiotics in RCTs against AD belong to *Lactobacilli* and *Bifidobacteria* genus, which are enriched in dairy products. These bacteria are able to modulate immune cells to restore the T_H_1/T_H_2 immune balance, enhance the production of the regulatory cytokine IL-10 and expand the population of Tregs [275], all of which benefit AD management. In addition, they compete with pathogenic bacteria, including *S. aureus* associated with AD, for nutrition and binding mucin [276]. Several RCTs attempted to test the efficacy of probiotics in preventing the development of AD (Table 3). Application of a single strain of probiotic demonstrates potent and enduring prevention against AD. Supplementation with *L. rhamnosus* GG (LGG) successfully reduced the incidence of AD in the first year of life by ~50% compared to placebo treatment in an RCT performed in Finland [277], and such protection extended to 4 years of age [278]. Another large-scale RCT in New Zealand suggested that early exposure to *L. rhamnosus* (HN001) had enduring protection against AD, at least in the first decade of life [279,280,281,282]. The further investigation suggested that the prevention against AD provided by HN001 may overcome the risk of AD caused by certain genetic deficiencies, particularly in genes encoding TLRs [283,284]. Nevertheless, supplements of HN001 did not have significant impacts on the infant gut microbiota [285]. A combination of various probiotics appears to be effective in preventing AD as well. An RCT performed in Norway demonstrated that maternal uptake of a combination of three probiotics, LGG, *L. acidophilus La-5* and *B. lactis* Bb-12, had a potent preventive effect against the development of AD up to 6 years, which is accompanied with a reduction in T_H_22 [286,287,288]. A probiotic mix comprised of *B. bifidum* BGN4, *B. animalis* subsp. *lactis* AD011 and *L. acidophilus* AD031 also significantly reduced the incidence of AD in the first year of life in a Korean RCT [289]. Supplement of *B. breve* and *B. longum* together exhibited similar benefits and reduced fecal *Proteobacteria* [290]. However, probiotic treatments, particularly prenatal treatments alone, failed to prevent the development of AD in early life in multiple other RCTs without comprehensive follow-ups [291,292,293,294,295]. Such variations may be due to the differences in the participants enrolled, experimental designs, cultures of the probiotics and the dose administered. A follow-up study from the Norwegian RCT [286,287,288] suggested that the efficacy of probiotics may depend on the intrinsic microbiota, that high levels of fecal *B. dentium* is associated with weaker protection against AD by the probiotics treatment [296]. Overall, systematic reviews and meta-analyses based on the literature above suggest that probiotics have a long-term preventive effect against AD [297,298,299,300,301,302,303,304,305,306,307,308,309,310,311]. Therefore, although contradictory results exist, WAO recommends using probiotics during pregnancy, lactation and infancy to prevent the development of allergy [312].

Supplementation with probiotics has also been used to treat AD in RCTs (Table 3). Many RCTs selected *L. rhamnosus*, particularly the strain LGG [277,278]. The first RCT using probiotics to manage AD achieved improvement in AD infants by orally applying LGG [313]. LGG alleviated infantile AD with no major impact on the gut microbiota in another RCT [314]. Nevertheless, LGG supplement failed to rescue infantile AD in multiple more recent RCTs [315,316,317]. Another strain of *L. rhamnosus*, MP108, showed a therapeutic effect on AD [318]. More recently, a mixture of three strains of *L. rhamnosus* (ŁOCK 0900, ŁOCK 0908, ŁOCK 0918) significantly benefitted infants with AD [319]. Supplement of other *Lactobacilli* appeared effective in alleviating AD symptoms as well, including *L. salivarius* [320,321], *L. fermentum* [322,323], *L. sakei* [324], *L. plantarum* [325,326,327], and *L. paracasei* [323], although contradictory results exist for *L. paracasei* [328]. Combination treatment of different *Lactobacilli* also potently attenuate AD [323,329,330]. *Bifidobacteria* are also used in RCTs against AD, particularly *B. lactis*. *B. lactis* Bb-12 was effective in improving AD in the first trial using probiotics to manage AD [313]. Supplementing *B. lactis* LKM512 expanded *Lactobacilli* in gut microbiota and alleviated AD, likely through the increased production of kynurenic acids, a product of tryptophan metabolism [331]. However, *B. lactis* CNCM I-3446 demonstrated no benefit in the treatment of AD [328]. Using the mixtures of *Lactobacilli* and *Bifidobacteria* appropriately may be a promising therapy against AD. Supplements of *L. salivarius* and *B. breve* combination conferred a significant improvement in adult AD patients and a potent regulation on the T_H_1/T_H_2 immune balance [332]. A mixture of *B. lactis*, *B. longum* and *L. casei* accelerated the recovery of moderate AD with steroid treatment [333]. Another combination of probiotics, *L. salivarius* and *S. thermophilus* is also beneficial for AD management [334]. Probiotic treatments selectively attenuated IgE-sensitized AD [335,336] and food-sensitized AD infants [337], suggesting probiotics may not be a wide-spectral therapy against AD. Aligned with the mixed results, systematic reviews and meta-analyses cannot conclude the efficacy of probiotics in treating AD [299,311,338,339,340,341,342,343].

While future RCTs may continue to identify novel species or strains of probiotics that may be effective in controlling and treating AD, more effort is needed to standardize the current probiotics applications in the battle against AD and to set up regulations in the use of probiotics.

**Table 3:** Summary of the randomized clinical trials using probiotics to prevent or treat AD.

Year	Completed Participants	Probiotics Used	Intervention Route and Duration	Results	Reference
**Prevention**					
2001	132 children with high risk of allergy and their mothers; 64 in probiotic group vs. 68 in placebo group	*L. rhamnosus* GG (ATCC 53103)	Oral/2–4 weeks prenatally + 6 months postnatally (by either mothers or infants)	Probiotic group: ↓incidence of AD in the first year of life and at 4 years of age	[277,278]
2007	178 children with atopic mothers; 89 in probiotic group vs. 89 in placebo group	*L. acidophilus* (LAVRI-A1)	Oral/the first 6 months of life	Probiotic group: no effect on AD in the first year of life, ↑incidence of allergen sensitization	[291]
2008	94 children with high risk of allergy and their mothers; 50 in probiotic group vs. 44 in placebo group	LGG	Oral/2–4 weeks prenatally + 6 months postnatally (by either mothers or infants)	Probiotic group: no effect on AD at 2 years of age, ↑incidence of recurrent wheezing bronchitis	[292]
2008	474 children with high risk of allergy and their mothers; 157 in HN001 group vs. 158 in HN019 group vs. 159 in placebo group	*L. rhamnosus* HN001 or *B. animalis* HN019	Oral/From 35 weeks gestation until 2 years postnatally (by mothers and children)	HN001 group: ↓incidence of AD at 2 years, 4 years, 6 years, and 11 years of age; HN019 group: no effect	[279,280,281,282]
2009	245 Asian infants with high risk of allergy; 124 in probiotics group vs. 121 in placebo group	*Bifidobacterium longum* BL999, *L. rhamnosus*	Oral/The first 6 months of life	No effect at 1 year of age	[293]
2010	112 children with high risk of allergy and their mothers; 57 in probiotics group vs. 55 in placebo group	*B. bifidum* BGN4, *B. animalis* subsp. *lactis* (*B. lactis*) AD011, *L. acidophilus* AD031	Oral/4–8 weeks prenatally + 6 months postnatally (by either mothers or infants)	Probiotics group: ↓incidence of AD in the first year of life	[289]
2010	278 children and their mother; 138 in probiotics group vs. 140 in placebo group	Combination of LGG, *L. acidophilus La-5*, *B. lactis* Bb-12	Oral/From 36 weeks of gestation until 3 months postnatally during breastfeeding by mothers	Probiotics group: ↓incidence of AD at 2 years and 6 years of age, ↓T_H_22, No adverse effect	[286,287,288]
2011	250 children with high risk of allergy and their mothers; 125 in probiotic group vs. 125 in placebo group	LGG	Oral/From 36 weeks of gestation until delivery	Probiotic group: no effect on AD in the first year of life, ↓CD14 and IgA in maternal breast milk	[294]
2014	158 children and their mothers; 122 in probiotics group vs. 36 in placebo group	Combination of *B. breve* M-16V, *B. longum* BB536	Oral/1 month prenatally + 6 months postnatally by infants	Probiotics group: ↓incidence of AD at 10 and 18 months of age, ↓fecal Proteobacteria, no adverse effect	[290]
2018	423 children with high risk of allergy and their mothers; 212 in HN001 group vs. 211 in placebo group	*L. rhamnosus* HN001	Oral/From 14–16 weeks of gestation until 6 years postnatally during breastfeeding by mothers	No effect on infantile AD	[295]
**Treatment**					
2000	27 infants with AD; 9 in LGG group vs. 9 in Bb-12 group vs. 9 in placebo group	LGG, *B. lactis* Bb-12 (Bb-12)	Oral/3 months	Probiotic groups: ↓AD symptoms, ↓serum soluble CD4, ↓urine eosinophilic protein X	[313]
2003	35 infants with AD; 14 in LGG group vs. 13 in heat-inactivated LGG group vs. 8 in placebo group	LGG	Oral/7.5 weeks	LGG group: ↓AD symptoms	[314]
2003	43 children with AD; 20 in placebo→probiotics, 23 in probiotics→placebo	Mixture of *L. rhamnosus* 19070-2 and *L. reuteri* DSM 122460	Oral/First intervention (6 weeks)→Washout (6 weeks)→Second intervention (6 weeks)	Probiotics treatment: ↓AD symptoms, ↓serum eosinophil cationic proteins	[329]
2004	80 in LGG group, 76 in mix group, 74 in placebo group	LGG, Mixture of 4 probiotics (Mix, LGG, *L. rhamnosus* LC705, *B. breve* Bbi99, *Propionibacterium freudenreichii* ssp. *Shermanii* JS)	Oral/4 weeks	Generally, no obvious effect, Probiotics group: ↓IgE sensitized AD	[336]
2005	53 children with moderate-severe AD (Topical corticosteroids were permitted); 26 in probiotic group vs. 27 in placebo group	*L. fermentum* VRI-003 PCC	Oral/8 weeks	Probiotics treatment: ↓AD symptoms	[322]
2006	59 children with AD; 29 in probiotics group vs. 30 in placebo group	Mixture of LGG and *B. lactis* Bb-12 (Bb-12)	Oral/18 weeks	All participants probiotics: ↓AD symptoms (non-significant); within food sensitized participants, probiotics group: ↓AD symptoms (significant)	[337]
2006	50 infants with AD; 17 in Lrh group, 16 in LGG group, 17 in placebo group	*L. rhamnosus* (Lrh), LGG	Oral/3 months	No therapeutic effect and no immune difference	[315]
2006	53 infants with moderate-severe AD (Emollients, class I–II topical corticosteroids and antihistamines were permitted); 26 in probiotic group, 27 in placebo group	LGG	Oral/8 weeks	No therapeutic effect	[316]
2007	102 infants with mild-moderate AD; 54 in probiotic group, 48 in placebo group	LGG	Oral/12 weeks	No therapeutic effect	[317]
2010	88 children with AD; 45 in probiotic group vs. 43 in placebo group	*L. sakei* KCTC 10755BP	Oral/12 weeks	Probiotic group: ↓AD symptoms, ↓serum CCL17 and CCL27	[324]
2011	141 children with AD; 45 in LP group, 47 in BL group, 47 in placebo group	*L. paracasei* CNCM I-2116(LP), *B. lactis* CNCM I-3446 (BL)	Oral/3 months	No therapeutic effect	[328]
2011	38 adult AD patients; 19 in probiotic group vs. 19 in placebo group	*L. salivarius* LS01 (DSM 22775)	Oral/16 weeks	Probiotic group: ↓AD symptoms, ↓fecal load of *Staphylococci*, ↓plasma LPS, enduring restoration of T_H_1/T_H_2 immune balance	[320,321]
2012	46 adult AD patients; 31 in probiotics group vs. 15 in placebo group	The combination of *L. salivarius* LS01, *B. breve* BR3	Oral/12 weeks	Probiotics group: ↓AD symptoms, ↓plasma LPS, ↓activated T cells, ↑T_H_1, ↓T_H_2, ↓T_H_17, ↑ Treg cells, ↓fecal *Staphylococci*	[332]
2012	118 children with AD (Emollients were permitted); 58 in probiotic group vs. 60 in placebo group	*L. plantarum* CJLP133	Oral/12 weeks	Probiotic group: ↓AD symptoms, ↓total eosinophil count, ↓IL-4 and IFNγ in blood	[326]
2014	25 adult AD patients; 13 in probiotics group vs. 12 in placebo group	*L. salivarius* LS01 (DSM 22775), *S. thermophilus* ST10 (DSM25246)	Oral/1 month	Probiotic group: ↓AD symptoms	[334]
2014	44 adult AD patients (Medications without probiotic effect and corticosteroid application were permitted); 22 in probiotic group vs. 22 in placebo group	*B. lactis* LKM512	Oral/8 weeks	Probiotic group: ↓AD symptoms, ↑fecal Lactobacilli, ↑fecal kynurenic acid	[331]
2015	212 children with moderate-severe AD; 55 in LP group vs. 53 in LF group vs. 51 in LP + LF group vs. 53 in placebo group	*L. paracasei* (LP), *L. fermentum* (LF) and the combination of LP and LF	Oral/3 months	LP group, LF group and LP + LF group: ↓AD symptoms, ↓serum IL-4, IgE, TNF, ↑serum IFN, TGF, ↓urine eosinophilic protein X, 8-OHdG	[323]
2017	62 children with AD; 30 in probiotic group vs. 32 in placebo group	*L. rhamnosus* (MP108)	Oral/8 weeks	Probiotic group: ↓AD symptoms	[318]
2017	22 children with AD; 12 in probiotic group vs. 10 in placebo group	*L. plantarun* IS-10506	Oral/12 weeks	Probiotic group: ↓AD symptoms, ↓serum IL-4, IFNγ, IL-17, ↑serum IL-10↑, ↑Treg cells in blood	[325]
2018	50 children with moderate AD who were prescribed topical steroids; 26 in probiotics group vs. 24 in placebo group	Mixture of *B. lactis* CECT 8145, *B. longum* CECT 7347, *L. casei* CECT 9104	Oral/12 weeks	Probiotics group: ↓AD symptoms, ↓steroids treatment	[333]
2020	109 adult AD patients; 29 in CCFM16 group vs. 43 in CCFM8610 group vs. 11 in oligosaccharide group vs. 26 in placebo group	*B. bifidum* CCFM16, *L. plantarum* CCFM8610	Oral/8 weeks	*L. plantarum* CCFM8610 group: ↓AD symptoms, ↑serum IL-10, ↓microbial functional genes involving *S. aureus* infection AD symptoms, ↑steroid hormone biosynthesis	[327]
2020	82 children with mild-moderate AD; 41 in probiotic group vs. 41 in placebo group	*L. pentosus*	Oral/12 weeks	Generally, no obvious effect, probiotic group: ↓IgE sensitized AD, (no difference in cytokine levels and microbial diversities)	[335]
2021	134 children with AD; 66 in probiotics group vs. 68 in placebo group	*L. rhamnosus* ŁOCK 0900, ŁOCK 0908, ŁOCK 0918	Oral/3 months	Probiotics group: ↓AD symptoms	[319]
2021	80 adult AD patients; 40 in Probiotics group vs. 40 in placebo group	*L. plantarum* PBS067, *L. reuteri* PBS072 and *L. rhamnosus* LRH020	Oral/56 days	Probiotics group: ↓AD symptoms, ↓skin TNF and TSLP	[330]

### 8.4. Synbiotics and Postbiotics in Treating AD

There are attempts to combat AD by combining the administration of prebiotics and probiotics. A new term “synbiotic” was defined in 2019 as “a mixture comprising live microorganisms and substrate(s) selectively utilized by host microorganisms that confers a health benefit on the host” [344]. RCTs against AD using synbiotics with limited scales demonstrated mixed results [264,345,346,347,348,349,350,351]. Nevertheless, well-designed synbiotics are likely to lead to novel, effective therapeutics against AD.

Postbiotics are inanimate microorganisms and/or their components with beneficial effects on host health [352], including the lysates of microorganisms, heat-inactivated microorganisms, and microbial metabolites. The trials using lysates and heat-inactivated microorganisms are highly contradictory. Early attempts using heat-inactivated LGG failed to benefit AD and even resulted in adverse gastrointestinal symptoms, including diarrhea [314]. Similarly, heat-treated *L. paracei* GM-080 did not accelerate the recovery of infantile AD with topical corticosteroid treatment [353]. On the contrary, trials using the postbiotics from the different strains of the same species were potent in improving AD. Tyndallized *L. rhamnosus* IDCC3201 (RHT3201) had therapeutic improvement in children with moderate AD and decreased eosinophil cationic protein and IL-31 in blood [354]. Heat-killed *L. paracasei* K71 alleviated adult AD and reduced the use of topical corticosteroids [355]. Heat-killed *L. acidophilus* strain L-92 attenuated AD symptoms in both children and adults [356,357], decreased eosinophil count, and increased serum TGF-β [357]. In addition, lysate of *Vitreoscilla filiformis* significantly improved AD and reduced fecal *S. aureus* [358]. The application of lysates and heat-inactivated microorganisms faces similar challenges as the application of probiotics in lacking standard and consistency.

Microbial metabolites have advantages in standardization, but current investigations are still limited to the pre-clinical stage [359]. With sufficient knowledge from pre-clinical studies, more trials should utilize microbial metabolites, including SCFAs, D-tryptophan, and IAld to test their clinical efficacy against AD.

## 9. Concluding Remarks

The recent discoveries on how the skin and gut microbiota modulate AD pathogenesis point out a bright potential in manipulating commensal bacteria to prevent and manage AD. Current therapies that leverage the skin microbiota, however, are still limited. Further investigations are needed to improve the specificity of the therapies against pathogenic bacteria, such as *S. aureus*, while encouraging the growth of normal bacteria at the same time. Clinical trials have been conducted to manipulate the gut microbiota for the prevention and treatment against AD. Although the efficacies of certain treatments are still controversial, the oral application of probiotics during pregnancy, lactation, and infancy is recommended for the prevention of allergic diseases, including AD [312]. More efforts are needed to standardize gut microbiota-based interventions against AD. In addition, microbial metabolites may aid in AD management, but they need to be tested in future trials. Furthermore, using these microbiota-based treatments in conjunction with the traditional treatments may achieve improved clinical outcomes. The role of commensal bacteria in AD is still a relatively new area. Increased understanding in this area would improve the management of AD, an increasingly prevalent atopic disease.

## Figures and Tables

**Table 2:** Summary of clinical studies demonstrating the alternation of gut microbiota in patients with AD.

Year	Subjects, Numbers	Methods	Results (Alternations of Gut Microbiota)	Reference
1999	Two-year aged children; 13 Estonian and 14 Swedish allergic subjects; 16 Estonian and 19 Swedish nonallergic subjects	Culture	Allergic subjects: ↑Aerobic bacteria, ↑Coliforms, ↑*S. aureus*	[143]
2001	22 atopic infants and 54 nonatopic infants	Culture, FISH	Atopic infants: ↑Clostridia	[163]
2001	Two-year aged children; 9 Estonian and 9 Swedish AD/allergic subjects; 13 Estonian and 11 Swedish healthy subjects	Culture	AD/allergic subjects: ↓Enterococci, ↓*Bifidobacteria*, ↓*Bacteroides*, ↑Clostridia, ↑*S. aureus*	[144]
2003	30 AD patients and 68 healthy individuals	Culture	AD patients: ↓*Bifidobacteria*, ↑*Staphylococci*	[164]
2006	21 AD toddlers and 28 healthy toddlers	Culture, FISH, quantitative flow cytometry, 16S rRNA gene sequencing	AD toddlers: ↓*Bifidobacteria*, ↑Clostridia, ↑Lactic producing bacteria, ↑*Enterococci*	[165]
2006	26 AD infants and 52 healthy infants	PCR, 16S rRNA DGGE profile	Infants who developed AD within the first year of life: ↑*E. coli*	[147]
2007	10 allergic infants and 16 healthy infants	16S rRNA gene sequencing	Allergic infants: ↑*Bifidobacterium catenulatum*, ↑*Bifidobacterium bifidum*	[167]
2007	324 infants	Culture	AD infants: ↑*S. aureus*, ↑*Bacteroides*	[145]
2007	957 infants with high risk of allergic diseases	Real-time PCR	Subjects with higher risk of AD: ↑*E. coli*, ↑*Clostridioides difficile*	[148]
2008	37 AD infants and 24 heathy infants	TTGE, FISH	AD infants: ↑*Bifidobacterium pseudocatenulatum*	[168]
2008	9 AD infants and 12 healthy infants	DGGE	AD infants: ↓-diversity	[130]
2008	15 AD infants and 20 healthy infants	T-RFLP, TTGE	AD infants: ↓-diversity	[131]
2010	19 AD infants and 22 healthy infants	16S rRNA gene sequencing	AD infants: ↓*Bifidobacteria*, ↓*Enterobacteriaceae*	[166]
2011	3303 children	Real-time PCR	AD children: ↑*Clostridioides difficile*	[149]
2011	411 infants with high risk of allergic diseases	PCR, 16S rRNA DGGE profile	↓-diversity	[132]
2012	20 AD infants and 20 healthy infants	16S rRNA gene sequencing	AD infants: ↓-diversity, ↑*Enterococcus* spp., ↑*Peptostreptococcaceae Incertae Sedis*, ↑*Eggerthella* spp., ↑*Coprobacillus* spp., ↑*Peptoniphilus* spp., ↓*Sutterela* spp., ↓*Fusobacterium* spp.	[133]
2012	33 AD infants and 65 healthy infants	T-RFLP	AD infants: ↓-diversity	[134]
2013	1402 infants	Real-time PCR	AD infants: ↑Clostridia	[150]
2015	28 AD infants and 11 healthy infants	HITChip	AD infants: ↓-diversity, ↓Butyrate-producing bacteria, ↓*Coprococcus eutactus*	[135]
2016	298 neonates	16S rRNA gene sequencing	Childhood AD history: ↓*Bifidobacteria*, ↓*Akkermansias*, ↓*Faecalibacteria*	[170]
2016	19 AD infants and 14 healthy infants	16S rRNA gene sequencing	AD infants: ↑*Bacteroidaceae*, ↑*Deinococcaceae*, ↓*Clostridiaceae*, ↓*Veillonellaceae*, ↓*Lactobacillaceae*	[140]
2016	50 AD infants and 51 healthy infants	16S rRNA gene sequencing	AD infants: ↑*Escherichia/Shigella*, ↑*Veillonella,* ↑*Faecalibacterium*, ↑*Lachnospiraceae incertae sedis*, ↑*Clostridium XIVa*, ↑*Faecalibacterium prausnitzii*, ↑*Ruminococcus gnavus*, ↑*Akkermansia muciniphila*, ↓*Bifidobacterium*, *Megasphaera*↓, ↓*Haemophilus*, ↓*Streptococcus*, ↓*Bacteroides fragilis*, ↓*Streptococcus salivarius*	[151]
2016	90 AD patients and 42 healthy subjects	16S rRNA gene sequencing and metagenomic sequencing	AD patients: A subspecies with of ↑*Faecalibacterium prausnitzii*, which is deficient in producing SCFAs, ↑Metabolic pathways responsive to oxidative stress↑, ↑Microbial genes encoding various transition metal transporters, ↑Microbial genes damaging mucin	[139]
2016	12 AD infants and 12 healthy infants	16S rRNA gene sequencing	AD infants: ↑Clostridia	[152]
2018	63 AD infants and 66 healthy infants	Metagenomic sequencing and real-time PCR	AD infants: Microbial genes associated with PI3K-Akt signalling, estrogen signalling, NOD-like receptor signalling, ↑antigen processing and presentation	[141]
2020	105 children	Metagenomic sequencing	AD children: ↓Microbial genes encoding carbohydrate active enzymes (CAZymes) from SCFA producers	[142]
2020	81 AD children and 58 healthy children	Real-time PCR	AD children: ↑*Clostridioides difficile*, ↑*Bifidobacteria*, ↓*Lactobacilli*, ↓*Eubacteria,* ↓*Lactobacillus* spp., ↓*B. fragilis*, ↓*E. coli*, ↓*Methanobrevibacter smithii*	[146]
2021	44 AD patients and 49 healthy subjects	16S rRNA gene sequencing	AD patients: ↓α-diversity, ↑*Porphyromonadaceae*↑, *Blautia*, ↑*Parabacteroides*, ↑*Bacteroides ovatus*, ↑*Bacteroides uniformis*, ↑*Prevotella stercorea*, ↓*Clostridium*, ↓*P. stercorea*	[137]
2021	1440 children	16S rRNA gene sequencing	AD children: ↓α-diversity, ↓*Lachnospiraceae*, ↓*Ruminococcaceae*_UCG-005, ↓*Christensenellaceae*_R-7_group spp.	[138]

Legend: FISH, fluorescence in situ hybridization; AD, atopic dermatitis; DGGE, denaturing gradient gel electrophoresis; TTGE, temporal temperature gel electrophoresis; T-RFLP, terminal restriction fragment length polymorphism; HITChip, the phylogenetic Human Intestinal Tract chip; PI3K, phosphatidylinositol 3-kinase; NOD, nucleotide-binding domain.
